# Supplementary material for: Comparative genomics provides new insights into the diversity, physiology, and sexuality of the only industrially exploited tremellomycete: Phaffia rhodozyma
Source: BMC Genomics. 2016 Nov 9;17:901. doi: 10.1186/s12864-016-3244-7 (PMC5103461; doi:10.1186/s12864-016-3244-7)
Supplement: Additional file 6: — List of orphan genes with links to PFAM (related to Additional file 1: Table S1). (ZIP 1428 kb) [file 12864_2016_3244_MOESM6_ESM.zip › PFAM_UNIQUE/G03849_P.html]

#                                                                            --- full sequence --- -------------- this domain -------------   hmm coord   ali coord   env coord
  
# target name        accession   tlen query name           accession   qlen   E-value  score  bias   #  of  c-Evalue  i-Evalue  score  bias  from    to  from    to  from    to  acc description of target
  
#------------------- ---------- ----- -------------------- ---------- ----- --------- ------ ----- --- --- --------- --------- ------ ----- ----- ----- ----- ----- ----- ----- ---- ---------------------
  
G03849\_P             -            336 PNRC                 PF15365.1     58    0.0019   16.9   0.0   1   2         4     6e+03   -5.0   5.4    15    33   156   175   150   220 0.69 - Proline-rich nuclear receptor coactivator  
G03849\_P             -            336 PNRC                 PF15365.1     58    0.0019   16.9   0.0   2   2   1.2e-06    0.0019   16.9   0.0     4    24   311   331   309   334 0.92 - Proline-rich nuclear receptor coactivator
